# Accomplishing a N-E-W (nutrient-energy-water) synergy in a bioelectrochemical nitritation-anammox process

**DOI:** 10.1038/s41598-019-45620-2

**Published:** 2019-06-24

**Authors:** Umesh Ghimire, Veera Gnaneswar Gude

**Affiliations:** Department of Civil and Environmental Engineering, Mississippi State University, Mississippi State, MS 39762 USA

**Keywords:** Environmental sciences, Energy science and technology

## Abstract

This study reports an investigation of the concept, application and performance of a novel bioelectrochemical nitritation-anammox microbial desalination cell (MDC) for resource-efficient wastewater treatment and desalination. Two configurations of anammox MDCs (anaerobic-anammox cathode MDC (AnA_mox_MDC) and nitration-anammox cathode MDC (NiA_mox_MDC)) were compared with an air cathode MDC (CMDC), operated in fed-batch mode. Results from this study showed that the maximum power density produced by NiA_mox_MDC (1,007 mW/m^3^) was higher than that of AnA_mox_MDC (444 mW/m^3^) and CMDC (952 mW/m^3^). More than 92% of ammonium-nitrogen (NH_4_^+^-N) removal was achieved in NiA_mox_MDC, significantly higher than AnA_mox_MDC (84%) and CMDC (77%). The NiA_mox_MDC performed better than CMDC and AnA_mox_MDC in terms of power density, COD removal and salt removal in desalination chamber. In addition, cyclic voltammetry analysis of anammox cathode showed a redox peak centered at −140 mV Vs Ag/AgCl confirming the catalytic activity of anammox bacteria towards the electron transfer process. Further, net energy balance of the NiA_mox_MDC was the highest (NiA_mox_MDC-0.022 kWh/m^3^ >CMDC-0.019 kWh/m^3^ >AnA_mox_MDC-0.021 kWh/m^3^) among the three configurations. This study demonstrated, for the first time, a N-E-W synergy for resource-efficient wastewater treatment using nitritation-anammox process.

## Introduction

Water and wastewater infrastructure accounts for approximately 3–4 percent of national energy demand in the United States^1^. More than 50 percent of the supplied energy is used to meet the aeration demands for carbon and nitrogen oxidation processes in wastewater treatment^2^. This particular energy demand is anticipated to increase significantly in the near future as many wastewater treatment plants aim to meet the USEPA’s strict regulations for nitrogen discharge (1.0 mg/L ammonium nitrogen for discharge to surface water and 10 mg/L total nitrogen (TN) for discharge to soil^1^). Current treatment schemes utilize autotrophic nitrification followed by heterotrophic denitrification techniques for nitrogen removal, adding significant demands for aeration energy and supplemental carbon. There is a growing interest to develop energy-positive and resource-efficient processes that minimize energy consumption in conventional processes and nitritation-anammox process is considered as one of the most promising alternatives to conventional biological nitrogen removal process^3^.

The scientific rationale for the proposed nitritation-anammox process stems from the anammox reaction stoichiometry shown in Reaction R1. Strous *et al*.^4^ described the physical purification of anammox bacteria, which oxidize ammonium to nitrogen gas anaerobically, with nitrite (NO_2_^−^) as an electron acceptor and fixed carbon from CO_2_ as a sole carbon source for the growth of anammox biomass (CH_2_O_0.5_N_0.15_), making the organism an autotroph (see reaction R1).

R1 $$\begin{array}{c}{{{\rm{1NH}}}_{4}}^{+}+1{{{\rm{.32NO}}}_{2}}^{-}+0{{{\rm{.066HCO}}}_{3}}^{-}+0{{\rm{.13H}}}^{+}\to 0.26\,{{{\rm{NO}}}_{3}}^{-}\\ \,+\,0{{\rm{.06CH}}}_{2}{{\rm{O}}}_{0.5}{{\rm{N}}}_{0.15}+2{{\rm{.03H}}}_{2}{\rm{O}}+1{{\rm{.02N}}}_{2}\end{array}$$

This anaerobic oxidation reaction is thermodynamically more favorable than the aerobic ammonia oxidation reaction. The reported Gibb’s free energy value for the anammox process is −358 kJ mol^−1^ NH_4_^+^ ^5^, which is higher than the aerobic ammonia oxidation process (ΔG = −235 kJ mol^−1^ NH_4_^+^)^6^. The oxidation of ammonium occurs in the anammoxosome of anammox bacteria where hydroxylamine and ammonium combine to produce an intermediate compound called hydrazine (see reaction R2), which is an energy-rich compound providing energy source to the bacteria^7^.

R2 $${{\rm{NH}}}_{2}{\rm{OH}}+{{{\rm{NH}}}_{4}}^{+}\to {{\rm{N}}}_{2}{{\rm{H}}}_{4}+{{\rm{H}}}_{2}{\rm{O}}+{{\rm{H}}}^{+}$$

Hydrazine then undergoes oxidation to produce dinitrogen gas generating four electrons (reaction R3) equivalent for reducing nitrite to form hydroxylamine (NH_2_OH) at the cytoplasm of anammox bacteria^8^.

R3 $${{\rm{N}}}_{2}{{\rm{H}}}_{4}\to {{\rm{N}}}_{2}+{{\rm{4H}}}^{+}+{{\rm{4e}}}^{-}$$

Based on this concept, nitrite acts as an electron acceptor in the anammox process. Thus, anammox bacteria can be used as biocatalysts to accept electrons in the microbial desalination cell (MDC) process. In addition, biocathodes in MDCs allow for additional treatment functions while replacing expensive and toxic chemical additives^9–11^. MDC is an emerging bio-electro-chemical system, which has been developed to accomplish desalination of saline water, treatment of wastewater, and generation of electricity using ion exchange membranes (IEM) (anion and cation), all in a single system^12,13^. Bioelectrochemical process has been considered as a promising technological platform for recovering energy- and high-value chemical products from various waste sources using different configurations^14–16^.

**Figure 1:**
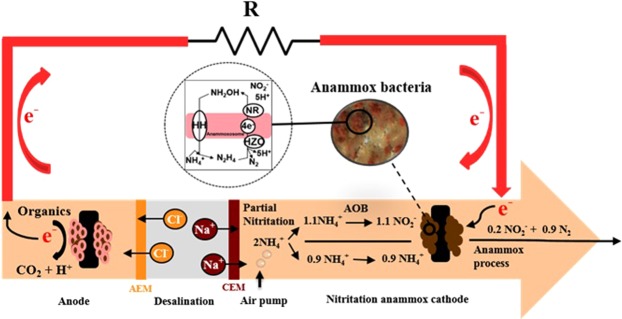
A N-E-W (Nutrient-Energy-Water) synergy in a bioelectrochemical nitritation-anammox process: anaerobic oxidation of organic matter takes place in anode compartment with release of electrons to be transferred to the partial nitritation-anammox process in the cathode compartment for nitrogen removal, interconnected in a bioelectrochemical configuration. The ionic imbalance in anode and cathode compartments facilitates a biologically mediated desalination in the microbial desalination cell.

Recently, Kokabian *et al*.^17^ reported a proof-of-concept study to demonstrate that anammox bacteria can be used as biocatalysts in MDCs to provide bioelectrochemical removal of carbon and nitrogen compounds from wastewater with electricity generation. A maximum power density of 0.092 W/m^3^ and >90% NH_4_^+^-N removal was achieved with an initial NO_2_^−^ concentration of 100 mg/L in the anammox biocathode. However, even in high strength municipal wastewaters, nitrites are not usually found^18^ and treating real municipal wastewater at the cathode chamber of MDCs using anammox process could be problematic due to lack of nitrites in the wastewater. Therefore, introducing partial nitritation-anammox process in the cathode chamber of anammox MDCs will oxidize a certain portion of ammonium to nitrite (Fig. 1). The nitritation-anammox process integrates two separate processes. Firstly, ammonium is partially nitrified to nitrite and then nitrite is denitrified using the residual ammonium by the anammox bacteria. Initially, nitritation and anammox reactions were separately achieved in two different reactors^19^. Later, the combination of nitritation and anammox reaction in a single compartment was successfully accomplished by controlling influencing parameters like aeration, dissolved oxygen (DO) and nitrite concentrations^20,21^. The single-stage nitritation-anammox process delivered good performance in both biofilm and granular sludge systems^22–24^. Anammox bacteria are slow growers with a doubling time of nearly 11 days^25^ and it is very important for anammox process to retain their biomass. Thus, a biofilm based process provides sufficient biomass retention and favors the growth of anammox bacteria. Moreover, in a single chamber nitritation-anammox system, co-existence of both nitrifiers and anammox bacteria could occur due to the prevalence of oxygen and oxygen free zones created by multiple layers of the biofilm, where nitrifiers grow on the surface and anammox grow inside^26^, which may create anoxic conditions suitable for anammox bacteria within a biofilm. MDCs consist of two biofilms, where electrons produced from the anode biofilm will be used by the anammox biofilm at the cathode, favoring the nitritation-anammox process. In contrast to the traditional nitrification/denitrification process, the nitritation-anammox process requires 60% less oxygen with low biomass production in a resource-, and environment-friendly manner^27–29^. Consequently, this process has already been studied intensively in recent years both at laboratory and pilot scales for wastewater treatment^22,30–33^. Although the single-stage nitritation-anammox process has been well studied by many researchers, the feasibility of bioelectrochemical nitritation-anammox process to treat wastewater with simultaneous saline water desalination and electricity production has not been studied. In this study, we present the bioelectrochemical nitritation-anammox process as a promising strategy to develop resource-efficient wastewater treatment systems.

The concept, application, and performance of a novel bioelectrochemical nitritation-anammox process to accomplish a resource-efficient nutrient-energy-water synergy was investigated. Three MDCs (NiA_mox_MDC, CMDC, and AnA_mox_MDC) were operated for more than 1,100 hours with identical process configurations and reactor sizes. Nitritation-anammox MDC (NiA_mox_MDC) was compared with an air cathode MDC (CMDC, which served as a control unit with an abiotic cathode) and an anaerobic anammox MDC (AnA_mox_MDC) based on power production, nitrogen removal, salinity removal, columbic efficiency, charge transfer efficiency, and COD removal. Energy consumption and generation trends by each MDC were analyzed to determine whether or not a configuration is energy-negative or energy-positive or energy-neutral. In addition, electrochemical impedance spectroscopy test was performed to compare the ohmic and mass transfer resistances of NiA_mox_MDC with CMDC, and anaerobic AnA_mox_MDCs.

## Results and Discussions

### Current density and power generation profiles

**Figure 2:**
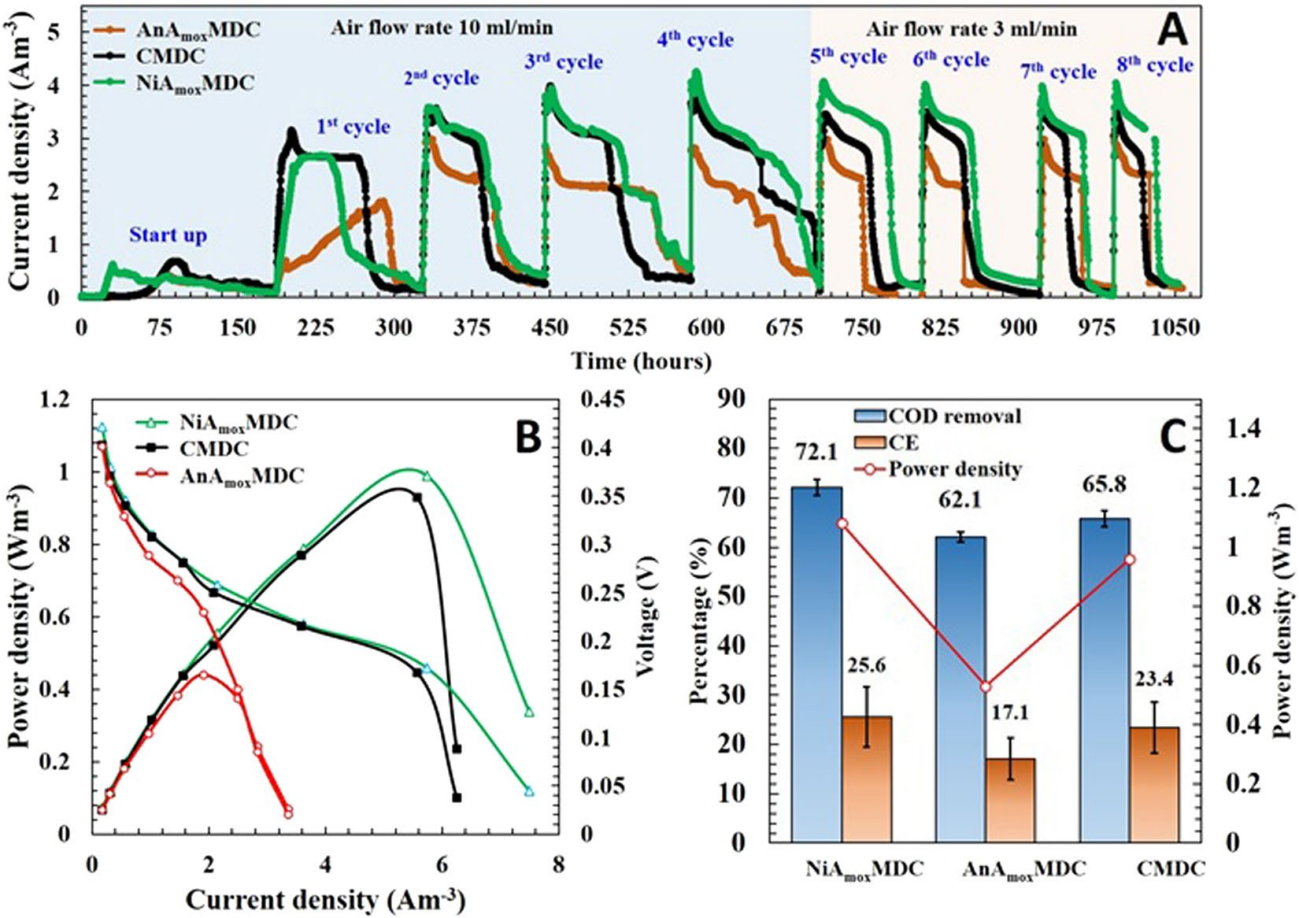
Comparison of performance: (**A**) Current density generated by different MDCs; (**B**) polarization and voltage profiles of different MDCs and (**C**) COD removal, CE and average power densities of different MDCs.

Three MDCs with varying cathode configurations (CMDC, AnA_mox_MDC and NiA_mox_MDC) were run in fed-batch mode to determine the maximum power density produced by these systems. The current density profiles of CMDC, AnA_mox_MDC, and NiA_mox_MDC were similar in all systems during the startup period, reaching a maximum current density of 0.65 A/m^3^, 0.39 A/m^3^, and 0.60 A/m^3^, respectively, across a 1-kΩ resistor. Startup times for CMDC and AnA_mox_MDC were longer than that of NiA_mox_MDC and their maximum current densities were erratic during the startup period (Fig. 2A). This characteristic behavior of MDCs was expected as it can be explained by the varying microbial community and population dynamics. The main reason is the inoculation of mixed-culture microbial community in the anode compartment, which often allows for non-anode respiring bacteria to occupy space on electrodes potentially limiting power generation and increasing startup time^34^. After this startup period, eight more fed-batch cycles were run in each MDCs. The maximum peak current density and maximum power density of CMDC and NiAmoxMDC increased with each subsequent fueling cycle (Supplementary Table S1). Maximum power density increased with continued fed-batch cycles, demonstrating a noticeable enhancement in the biocatalytic activity of the biofilm. These results indicated that the current density was improved mainly due to the formation of exoelectrogenic biofilms on the anode electrodes^17^. Polarization tests were conducted for each cycle as shown in Fig. 2B. NiA_mox_MDC generated the highest power density (1,007 mW/m^3^) with a decreasing order of CMDC (952 mW/m^3^) and AnA_mox_MDC (444 mW/m^3^). A possible reason for higher generation of power density in NiA_mox_MDC could be due to the presence of two terminal electron acceptors in a single cathode compartment: one reduction of oxygen and another metabolic activity of anammox bacteria. For example, electrons generated by exoelectrogenic bacteria via bio-catalyzed reactions (oxidation of organic substrate) at the anode were transferred to the cathode through an external electric circuit, where protons are combined with the electrons and oxygen to produce water (Fig. 1). In addition, in order to create the proton motive force (PMF) over the anammoxosomal membrane, nitrite (NO_2_^−^) is reduced to nitric oxide by accepting electrons, which then reacts with ammonium to produce hydrazine in anammox bacteria^7^. AnA_mox_MDC produced a maximum power density of 440 mW/m^3^, which was approximately 4.7 times higher than the previously reported value using anammox biofilm biocathode in a similar configuration (92 mW/m^3^)^17^.

### COD removal and Columbic Efficiency (CE)

COD removal rates in the anode chambers of CMDC, AnA_mox_MDC, and NiA_mox_MDC over the fed-batch cycles were 65.8 ± 1.56% (n = 8; SD = 1.56), 62.1 ± 1.07% (n = 8; SD = 1.07), and 72.1 ± 1.6% (n = 8; SD = 1.6), respectively (Fig. 2C) and their corresponding average CEs were 23.4 ± 5.2%, 17.1 ± 4.3, and 25.6 ± 6.1%, respectively (Fig. 2C). There were no statistically significant differences in the CEs between the three MDCs (ANOVA, p > 0.135). However, the COD removal efficiency was significantly higher in NiA_mox_MDC (ANOVA, p < 0.0001) (Supplementary Fig. S3). Higher power density in NiA_mox_MDC implies that a higher number of electrons were generated from the organic matter oxidation by the anode biofilm. As a result, the COD removal was higher in NiA_mox_MDC than the other MDCs. However, the CE of NiA_mox_MDC was only 25 ± 6.4%, indicating that a substantial portion of COD utilization was not associated with electron release. Relatively low CEs of all MDCs suggested that the organic matter oxidation may have been caused by other competing processes. Most of the MDC studies have used acetate, an easily biodegradable substrate, resulting in higher COD removals (mostly over 70%)^35,36^. In this study, glucose was used as an organic substrate and it is considered the most favorable substrate generating highest power density but lowest coulombic efficiencies when compared with other substrates^37^. Because it is a fermentable substrate, which implies its possible consumption by diverse competing metabolisms such as fermentation and methanogens that do not contribute towards columbic efficiency.

### Nitrite and ammonium removal in different MDCs

**Figure 3:**
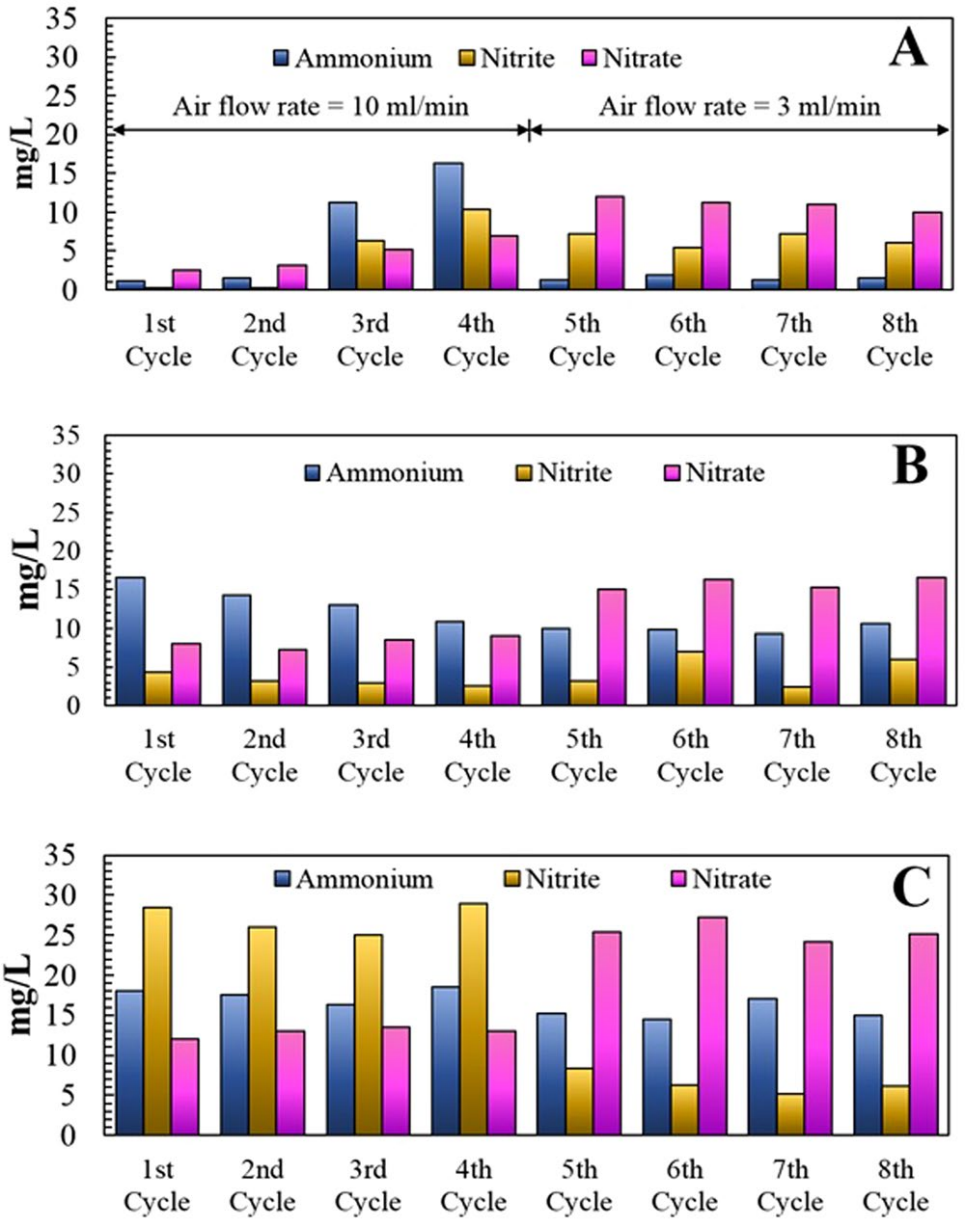
Ammonium, nitrite, and nitrate nitrogen concentrations at the end of each cycle: (**A**) in NiA_mox_MDC; (**B**) AnA_mox_MDC; and (**C**) CMDC.

Ammonium-nitrogen (NH_4_^+^-N) and nitrite-nitrogen (NO_2_^−^-N) removal rates were calculated. The cathode chamber of CMDC was fed with 70 mg/L NH_4_^+^-N at an air flowrate of 10 ml/min without anammox bacteria whereas the cathode chamber of NiA_mox_MDC was fed with 70 mg/L of NH_4_^+^-N along with enriched mixed-culture anammox bacteria at an air flow rate of 10 ml/min. To compare the nitrogen removal efficiency and power generation of NiA_mox_MDC, another MDC with an anaerobic anammox biocathode (AnA_mox_MDC) was operated, which was fed with 70 mg/L of NH_4_^+^-N and the same amount of NO_2_^−^-N under near-anaerobic conditions. Results showed that the NH_4_^+^-N removal rate in CMDC was almost constant in all four batch cycles (NH_4_^+^-N removal rates in four CMDC cycles were 75%, 75%, 77.3%, and 73.5%, respectively) (Supplementary Table S2). This trend confirmed oxidation of NH_4_^+^-N in the cathode chamber of CMDC. The removal of NH_4_^+^-N in CMDC cathode chamber could be due to the oxidation of ammonium-nitrogen to NO_2_^−^-N, NO_3_^−^-N or nitrogen gas. This was confirmed by analyzing the nitrite-nitrogen and nitrate-nitrogen concentrations in the cathode of CMDC at the end of each cycle. The results showed that at the end of first, second, third, and fourth cycles, the CMDC cathode compartment contained 27 mg/L, 26 mg/L, 25.3 mg/L, and 27.5 mg/L of NO_2_^−^-N and 12 mg/L, 13.5 mg/l, 14.3 mg/L, and 13.3 mg/L of NO_3_^−^-N respectively (Fig. 3C). This data indicated that a fraction of ammonium present in the cathode of CMDC was oxidized to nitrite and nitrate and the remainder of ammonium nitrogen could have been converted to nitrogen gas. Therefore, from the above results, it can be concluded that, in the cathode chamber of CMDC, oxygen acted as a terminal electron acceptor and oxidized NH_4_^+^-N to form NO_2_^−^-N, NO_3_^−^-N.

Cathode chamber of NiA_mox_MDC was fed with 70 mg/L of NH_4_^+^-N along with enriched mixed cultured anammox bacteria and aeration of 10 ml/min. The only difference between CMDC and NiA_mox_MDC was CMDC cathode lacks anammox bacteria whereas NiA_mox_MDC cathode was inoculated with anammox bacteria under identical conditions. The purpose of this test is to understand the applicability of nitritation-anammox process in MDC cathode. The concentrations of NH_4_^+^-N, NO_2_^−^-N, and NO_3_^−^-N were measured before and after each batch cycles of NiA_mox_MDC. The results showed that in NiA_mox_MDC, nitrite was not detected and NH_4_^+^-N removal was 98.3% and 97.8% (Supplementary Table S2) at the end of first and second batch cycles, respectively, but at the end of the third and fourth cycles, accumulation of nitrite was observed and the removal rate of NH_4_^+^-N was decreased to 84.8% and 78.6%, (Supplementary Table S2) respectively. The accumulated NO_2_^−^-N concentration at the end of the third and fourth cycles were 6.3 mg/L, 10.3 mg/L respectively (Fig. 3A). The absence of NO_2_^−^N and higher removal rate of NH_4_^+^-N (98.3% and 97. 9%) during first and second batch cycle confirmed the activity of annamox bacteria, which oxidize ammonium to nitrogen gas by nitritation-anammox process, with nitrite as an electron acceptor^25^. However, accumulation of NO_2_^−^-N and decrease in the removal rates of NH_4_^+^-N in third and fourth cycles confirmed the inhibition effect on the anammox process due to continued exposure of anammox bacteria to aerobic condition over a longer period. The inhibition of anammox process in nitritation-anammox due to aeration was reported by several researchers. Hu *et al*.^32^ reported that the activity of anammox bacteria decreased under aerobic conditions in the nitritation-anammox sequencing batch reactor. Similarly, Joss *et al*.^38^ also reported that a significant increase in the accumulated NO_2_^−^-N was observed at the end of the aeration phase of a combined nitritation-anammox process indicating significant inhibition of anammox process due to aeration. Another study also reported that the accumulation of nitrite increased to 2.54 mg/L under aerobic condition and after turning it to anoxic condition, the nitrite accumulation decreased from 2.54 mg/L to 1.37 mg/L^39^, reactivating the anammox process. Therefore, accumulation of nitrite at the end of the third and fourth cycles in NiA_mox_MDC strongly suggested the inhibition of anammox process.

Nitrogen removal rates of NiA_mox_MDC were compared with anaerobic anammox biocathode, AnA_mox_MDC. The cathode was maintained under anaerobic condition and the concentration of NO_2_^−^-N and NH_4_^+^-N were analyzed before and after the fed-batch cycle in the AnAmoxMDC. The results showed that the nitrite-nitrogen removal for the first, second, third, and fourth batch cycles were 41.9%, 47.4%, 70.9%, and 79.1%, respectively (with initial NO2^−^-N concentration of 70 mg/L)(Fig. 3B). Similarly, ammonium-nitrogen removal for the first, second, third, and fourth batch cycles were 76.7%, 79.5%, 81.6%, and 85.6%, respectively (Supplementary Table S2). This particular trend verified the improvement in the removal of nitrogenous compounds after several fed-batch cycles and the results are consistent with those of other studies^17^. The ammonium-nitrogen removal rates for the first, and second fed-batch cycles are significantly higher in NiA_mox_MDC than those of AnA_mox_MDC and CMDC. Furthermore, this finding also confirms the activity of annamox bacteria in the cathode of AnA_mox_MDC, which oxidized the ammonium to nitrogen gas anaerobically, with nitrite as an electron acceptor. After the end of fourth cycle, the air flow rates in CMDC and NiA_mox_MDC were reduced to 3 ml/min to maintain DO at 1.6 mg/L. All MDCs were run for one month at this air flow rate in four fed-batch cycles. After decreasing the air flow rate to 3 ml/min, 75% of the total nitrogen removal (Supplementary Fig. S1A) was observed in NiA_mox_MDC, which is 66.6% higher than the previous cycle, at an air flow rate of 10 ml/min. Supplementary Table S3 compares this process with other nitritation-anammox processes. It is clear that the bioelectrochemical nitritation-anammox process alone has the ability to provide an energy-yielding route to nitrogen removal.

### Desalination performance

**Figure 4:**
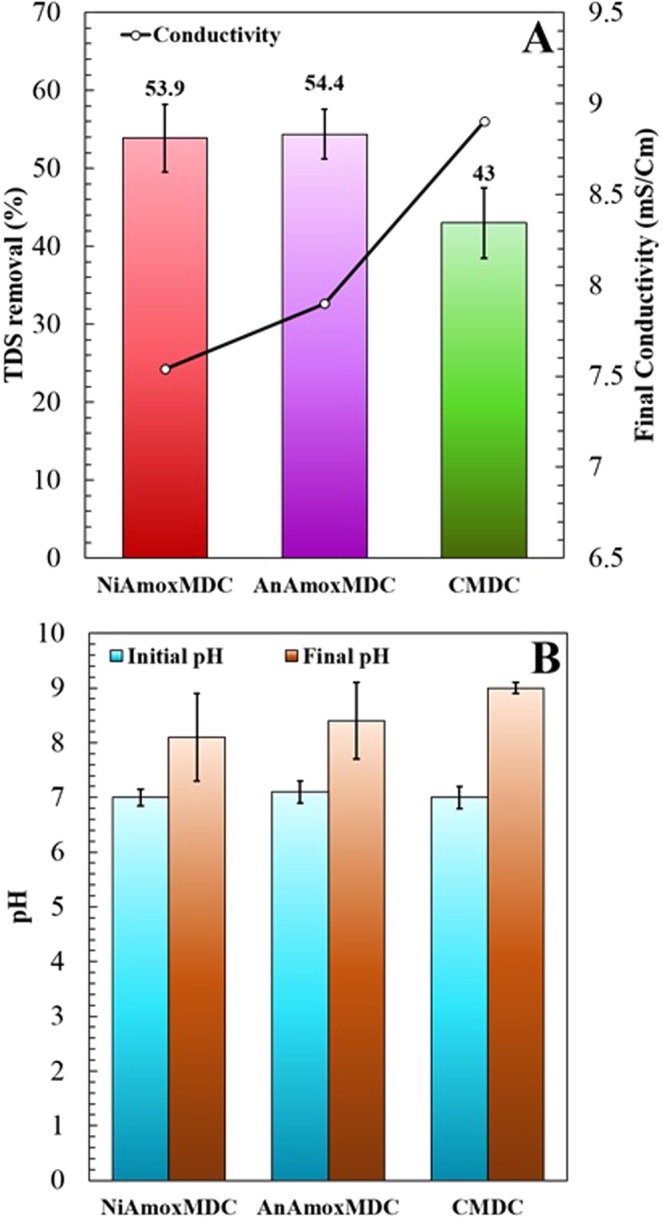
(**A**) Average TDS removal at the end of each cycle in MDCs, and (**B**) initial and final pH of cathode chamber in MDCs.

The total dissolved solids (TDS) concentrations in the middle compartment of three MDCs were measured at the end of each batch cycle. Figure 4A shows the average salinity removal rates for the three MDCs. Initially, 10 g/L of NaCl was fed in desalination compartment of each MDC. The electrical conductivity of 10 g/L of NaCl at 25 °C was 17.92 mS/Cm. The average salinity removal rates (TDS removal) in CMDC, AnA_mox_MDC and NiA_mox_MDC were 43.0, 54.4, and 53.9%, respectively (Fig. 4A). The percentage salinity removal was found to be nearly equal in NiA_mox_MDC and AnA_mox_MDC (53.9% and 54.4% respectively) but higher than that of CMDC (43%). The higher salinity removal in NiA_mox_MDC and AnA_mox_MDC than that of CMDC could be due to the presence of biocathode. It has been reported that the salinity removal is higher in biocathode MDC than in air cathodes^40,41^. The salinity removal in MDC is driven by the ionic imbalance caused by the electron-release-transfer-acceptance processes which allow for ion transfer from the desalination chamber to anode and cathode compartments. This process is also dependent on the volume of saline water, volume of wastewater, hydraulic retention time, wastewater concentration, membrane surface area, oxygen reduction, microbial oxidation and volumetric ratio (volume of anode/cathode compartments to the volume of desalination compartment)^42^. A higher volumetric ratio will facilitate a higher desalination rate. The volumetric ratio used in this study is 1.5 (v/v). At a higher volumetric ratio of 100 (v/v) with 35 mg/L of NaCl solution and potassium ferricyanide as catholyte, 93% salt removal was reported^43^. Similarly, 100% salinity removal was achieved at a volumetric ratio of 11 (v/v) with an air cathode and 30 mg/L of NaCl concentration^44^. Chen *et al*.^45^ reported 80% salinity removal at a volumetric ratio of 36 (v/v) in an air cathode MDC. Wen *et al*.^41^ used an MDC with a volumetric ratio of 11.3 (v/v) and obtained 92% of desalination rate with 35 mg/L of NaCl and a biocathode. However, at a volumetric ratio of less than 1, the salinity removal percentages reported were less than 50%. Mehanna *et al*.^35^ obtained a desalination rate of 37% with a volumetric ratio of 1 (equal volumes of anode, desalination and cathode chamber) and 44% of salinity removal was observed in an air cathode MDC at a volumetric ratio of 2 (with 35 mg/L of NaCl)^46^. Similarly, Zhang and He reported a salinity removal of about 57.8% in an osmotic MDC with a volumetric ratio of 0.8 (v/v)^47^. Therefore, the desalination rate in our study is consistent with other studies reported for similar volumetric ratios. In addition, a larger surface area of IEM would also improve ion transport and salt removal. In a study, Cao *et al*.^43^ reported 93% salinity removal with surface area of IEM and salt solution volume ratio of 3:1 (9 cm^2^:3 cm^3^), while this ratio in our MDC system was around 0.8:1 (31.8 cm^2^:40 cm^3^). Therefore, this study demonstrated the importance of MDC design factors such as volumetric ratio and IEM surface area on salinity removal, which should be taken into consideration in further evaluation of anammox MDCs. Further, pH of the cathode solution was recorded at the start and end of each fed-batch cycle for all MDCs. Figure 4B shows the average pH of cathode solution. Increase of pH of cathode solution at the end of each fed-batch cycle in the anammox process suggested hydrogen ion consumption linked to nitrate reduction and therefore acid neutralization^17,48^.

### Charge transfer efficiency

In order to further comprehend the desalination efficiency and its interaction with electricity generation, charge transfer efficiencies (CTE) of each MDCs were calculated and tabulated in Supplementary Table S4. The results showed that the CTE of NiA_mox_MDC was calculated as 588% (Supplementary Fig. S2) which was higher than that of AnAmoxMDC (550%) and CMDC (544%). The calculated average charge transfer efficiencies were above 100%, demonstrating that apart from electricity generation, other factors such as osmotic pressure and ion exchange process also contributed to salinity removal^35,44^. In our MDC, movement of water from one compartment to another compartment through the membranes was observed, which is caused by osmotic pressure differences caused by salinity differences in those compartments. When treating a 10 g/L of NaCl, the volume of desalinated water increased by 10% at the end of each cycle, which is due to the water movement from the anode chamber to the desalination chamber due to the osmotic pressure difference between the compartments. This movement of water to desalination chamber also contributed to the dilution of the salt solution, thus increasing charge transfer efficiency.

### Electrochemical performance

#### Cyclic Voltammetry (CV) test

**Figure 5:**
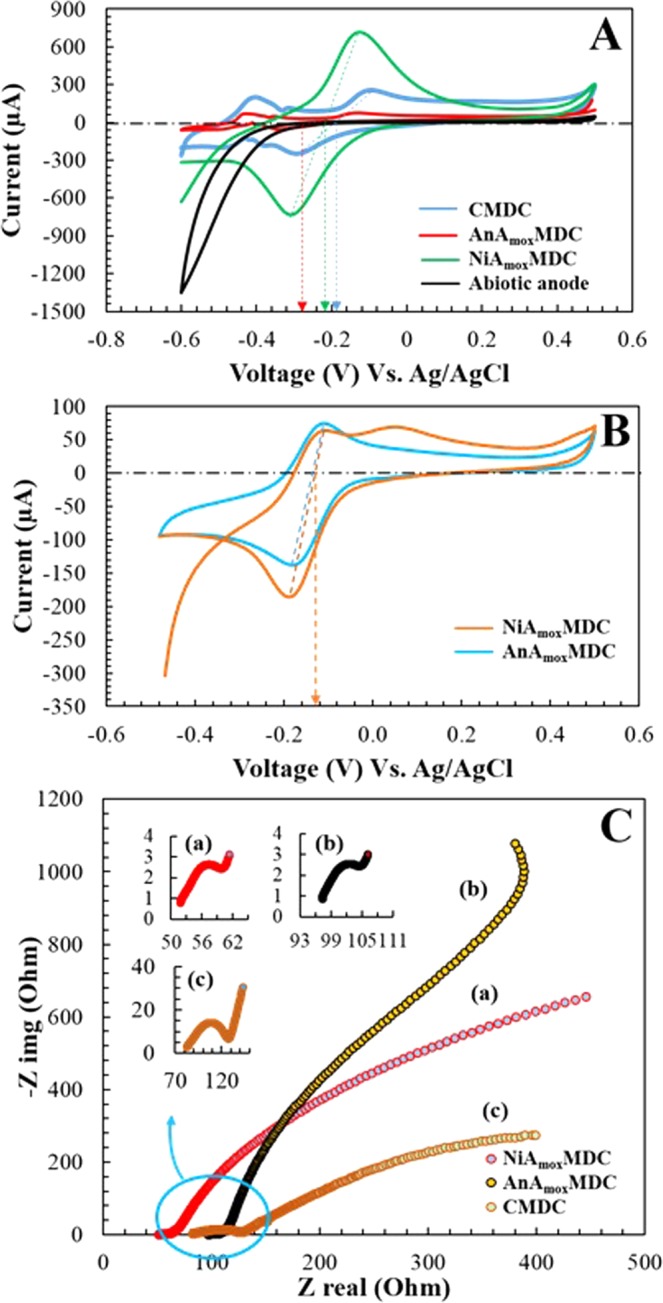
(**A**) Cyclic voltammograms of anode of AnA_mox_MDC, NiA_mox_MDC, abiotic anode and CMDC during turnover condition, (**B**) Cyclic voltammograms of biocathode of AnA_mox_MDC, and NiA_mox_MDC, and (**C**) Nyquist plots used to determine the ohmic resistance of AnA_mox_MDC, NiA_mox_MDC, and CMDC at the end of a batch cycle.

Anodic cyclic voltammetry (CV_anode_) test was performed at the turn over condition (a condition at which a stable voltage is observed in MDCs) of each MDCs. A comparison of the redox activities and biofilm performances of anodic biofilm in AnA_mox_MDC, NiA_mox_MDC and CMDC (Fig. 5A) was performed through CV tests. The results showed that NiA_mox_MDC produced higher oxidation current of 706 µA compared to that of CMDC (254 µA) and AnA_mox_MDC (77.7 µA) (Fig. 5A). The midpoint potential in NiA_mox_MDC, CMDC, and AnA_mox_MDC during the turnover condition was observed between −200 mV and −300 mV Vs Ag/AgCl, which is consistent with the measured midpoint potential for *G*. *Sulfurreducens*^49^. The larger size of cyclic voltammograms for NiA_mox_MDC than that of AnA_mox_MDC may indicate a better redox activity and biofilm performance at the anode of NiA_mox_MDC. No redox peaks were observed in CV test of the abiotic anode. This implies that microorganisms forming the biofilm at the biotic anode had a pronounced electrochemical activity when compared to an abiotic anode. Further, the redox activities and biofilm performances of cathodic biofilms (anammox biofilm) in AnA_mox_MDC and NiA_mox_MDC, were compared with cathode as active working electrode and anode as a counter electrode. The results showed that the observed redox peak was centered at −140 mV Vs Ag/AgCl for both AnA_mox_MDC and NiA_mox_MDC (Fig. 5B). The redox peak observed on CV_cathode_ analysis of anammox cathode confirmed the catalytic activity of anammox bacteria towards the electron transfer from cathode electrode material to anammox bacteria.

#### EIS test

In order to investigate the electrochemical performances and to determine the ohmic resistance of NiA_mox_MDC, CMDC, and AnA_mox_MDC, EIS was performed at an open circuit voltage of each MDC at the end of each cycle. EIS measurements indicated that the ohmic resistance of AnA_mox_MDC was 97.3 Ω, which is approximately two times higher than that of NiA_mox_MDC (50.1 Ω) at the end of the fed-batch cycle (Fig. 5C). The potential sources of energy loss are ohmic resistance, charge transfer resistance and diffusion resistance, of which, ohmic resistance is often the key factor in the performance of a MDC^50^. Higher ohmic resistance of AnA_mox_MDC caused a higher energy loss, which was consistent with its low performance in CV, voltage generation and polarization test. Luo *et al*.^51^ found that the ohmic resistance of an MDC increased from 98 Ω to 460 Ω after 5,300 h of operation, which was likely due to biofouling on the anion exchange membrane (AEM) caused by complex composition of the wastewater fed to the anode chamber and long-term operation of MDC^51^. In a similar study performed by Cao *et al*.^43^ ohmic resistance was found to increase by a factor of 40 (25 Ω at start to 970 Ω at the end) with salt concentration of 5 g/L in the desalination chamber^43^. Biofouling of both AEM and CEM may have caused a higher ohmic resistance in AnA_mox_MDC in this study as the cathode chamber of AnA_mox_MDC was maintained under anaerobic conditions. However, the cathode chamber of NiA_mox_MDC is provided with aeration and it has been reported that aeration minimizes biofouling^52^. Therefore, this study suggested that biofouling can have a significant effect on the performance of MDCs and further characterization of AEM- and CEM- biofouling is essential to improve the performance of biocathode MDCs.

#### Energy balance

**Figure 6:**
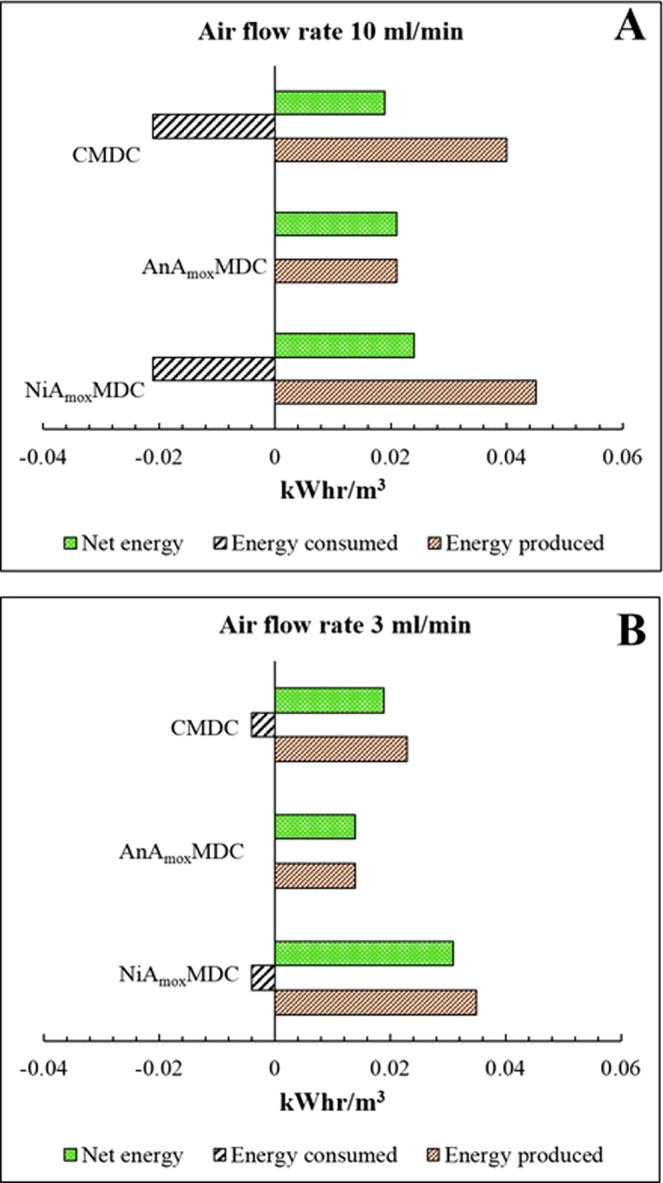
Energy production, energy consumption and net energy production of AnA_mox_MDC, CMDC and NiA_mox_MDC: (**A**) at a flow rate of 10 mL/min; and (**B**) at a flow rate of 3 mL/min.

Net energy production along with reduced energy consumption is the key to accomplish an energy-positive nutrient-energy-water synergy in microbial desalination cells. Energy consumption can be lowered by eliminating or by minimizing the aeration required in the cathode^53^. The energy production and consumption (expressed as kilowatt hour per cubic meter of treated water) trends in the three MDCs were compared in order to establish a preliminary energy balance (Supplementary Table S5). An MDC with net-positive (i.e., energy production – energy consumption) energy value was termed as energy-positive, while an MDC with net-negative energy value was termed as energy-negative. The results indicated that at the startup period, energy consumed by NiA_mox_MDC was 0.027 kWh/m^3^, which is nearly 300 times higher than the energy produced (0.0000879 kWh/m^3^). This condition was expected and is attributed to the poor startup condition. After the startup period, the first cycle was run and at the end of the first cycle, the energy produced by NiA_mox_MDC increased to 0.0221 kWh/m^3^ which was nearly equal to the energy consumed by aeration bringing the MDC to energy-neutral status (energy produced is equal to energy consumed). In the same cycle, AnA_mox_MDC produced 0.01 kWh/m^3^ net energy functioning as an energy-positive system and CMDC produced 0.036 kWh/m^3^ while consuming only 0.027 kWh/m^3^ for aeration. The net energy produced by NiA_mox_MDC in subsequent (second, third and fourth) cycles were higher than the net energy produced by CMDC and AnA_mox_MDC. The results also showed that the net energy production in NiA_mox_MDC increased with subsequent fueling cycle (Supplementary Table S5) whereas in the fourth cycle, the net energy production decreased in both CMDC and AnA_mox_MDC. An energy balance was also studied at air flow rate of 3 ml/min in NiA_mox_MDC and CMDC. The average energy production and consumption of three MDCs at air flow rates of 10 ml/min and 3 ml/min are shown in Fig. 6A,B, respectively. NiA_mox_MDC produced the highest net energy of 0.022 kWh/m^3^ followed by AnA_mox_MDC (0.021 kWh/m^3^) and CMDC (0.019 kWh/m^3^) at an air flow rate of 10 ml/min. However, when the air flow rate was reduced to 3 ml/min, the NiA_mox_MDC produced a net energy of 0.031 kWh/m^3^ (Fig. 6B) which is about 30% higher than the net energy produced at an air flow rate of 10 ml/min. Higher net energy production was achieved by reducing the aeration energy consumption (aeration energy consumption decreased by 170% when the air flow rate was reduced to 3 ml/min).

## Conclusions

This study has shown that a bioelectrochemical nitritation-anammox process can provide a resource-efficient ammonium-rich wastewater treatment. A maximum power density of 1,007 mW/m^3^ with more than 92% of ammonium removal was achieved in NiA_mox_MDC. Lower ohmic resistance of NiA_mox_MDC (50.1 Ω) than that of AnA_mox_MDC (97.3 Ω) indicated a higher electricity generation potential in NiA_mox_MDC when compared to AnA_mox_MDC. The findings of this research also showed that NiA_mox_MDC can be operated as an energy-positive MDC system, achieving a N-E-W synergy between nutrient, energy and water in bioelectrochemical systems. It is noted that exposing anammox bacteria to higher dissolved oxygen levels over a long period inhibited their activity due to the accumulation of NO_2_^−-^N and reduced NH_4_^+^-N removal rate in the cathode chamber. While this study demonstrated the performance of the bioelectrochemical nitritation-anammox process in a single cathode compartment, further studies should consider separating the nitritation and anammox compartments and transferring the wastewater from nitritation to anammox cathode compartment. This approach will maintain the anammox bacteria in relatively low dissolved oxygen concentrations to enhance the anammox activity and to optimize the bioelectrochemical nitritation-anammox process performance.

## Materials and Methods

### Anammox cultures

Anammox biomass was received from the HRSD York River wastewater treatment plant in Virginia and was cultured in an anaerobic batch reactor at a temperature of 35 °C. The anammox mineral media contained: (NH_4_)_2_SO_4_, 330 mg/L (5 mM); NaNO_2_, 345 mg/L (5 mM); KH_2_PO_4_, 27.2 mg/L; KHCO_3_, 500 mg/L; MgSO_4_.7H_2_O, 300 mg/L; CaCl_2_.2H_2_O, 180 mg/L, and trace element solutions I and II (1 ml/L). Trace element solution I contained: EDTA, 5 g/L; FeSO_4_, 5 g/L; and trace element solution II contained: EDTA, 15 g/L; ZnSO_4_.7H_2_O, 0.43 g/L; NaMoO_4_.2H_2_O, 0.22 g/L; NiCl_2_.6H_2_O, 0.19 g/L; CoC1_2_. 6H_2_O, 0.24 g/L; MnC1_2_. 4H_2_O, 0.99 g/L; CuSO_4_. 5H_2_O, 0.25 g/L; NaSeO_4_.10H_2_O, 0.21 g; and H_3_BO_4_, 0.014 g^54^. The anammox bacteria received from the HRSD York River wastewater treatment plant were acclimatized in a batch reactor for four months and during this process anammox media was replenished every three days. After four months, acclimated anammox biomass from the anammox reactor was initially inoculated in the cathode chamber of MDCs and operated in fed-batch mode.

### Anode inoculum

The anode compartments were inoculated with pre-acclimated aerobic sludge (fed with glucose for five months in a batch reactor) collected from the oxidation ditch of the Starkville wastewater treatment plant. The primary source of organic carbon fed to the anode of MDCs was glucose (468.7 mg/L). The anode compartment was fed with synthetic wastewater prepared with 0.13 g/L of KCl, and NH_4_Cl, 0.31 g/L nutrient solution in 50 mM phosphate buffer (NaH_2_PO_4_ ·H_2_O, 2.45 g/L, Na_2_HPO_4_, 4.58 g/L, pH = 7) and trace minerals^55^.

### MDC configuration and operation

**Figure 7:**
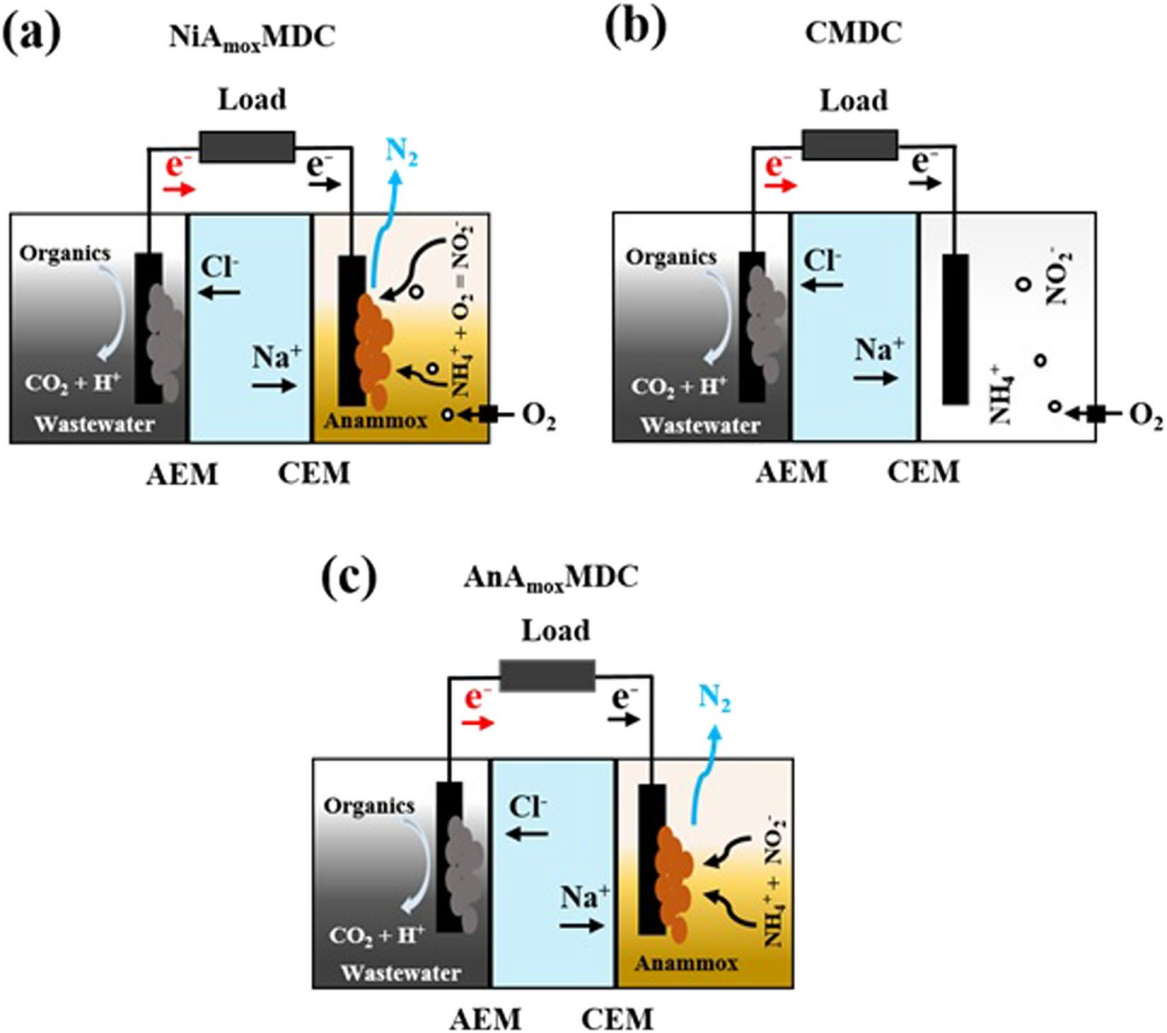
Schematic of experimental layouts for: (**a**) NiA_mox_MDC; (**b**) CMDC; and (**c**) AnAmoxMDC process configurations: NiA_mox_MDC, contained anammox bacteria as biocatalysts in the cathode compartment and air was supplied to achieve partial nitritation process. CMDC was operated as air cathode MDC, without anammox bacteria in cathode chamber and AnA_mox_MDC contained anammox bacteria as biocatalysts in the cathode compartment and was operated under near anaerobic conditions to perform direct anammox process in the cathode compartment.

In order to evaluate the role of anammox bacterium as a biocatalyst in the cathode chamber, three different MDCs were examined to compare the power density and energy generation profiles, and COD and nitrogen removal rates (Fig. 7). The three different MDCs were named: Nitritation-anammox MDC (NiA_mox_MDC), control MDC (CMCD), and anaerobic anammox MDC (AnA_mox_MDC). All three MDCs had similar process configurations and reactor sizes. Three-compartment MDCs were fabricated using plexi-glass (7.2 cm diameter) with an anode volume (V_an_) of 60 ml, a cathode volume (V_ca_) of 60 ml, and a desalination volume (V_ds_) of 40 ml. Anode and desalination compartments were separated by an anion exchange membrane (AMI7001, Membranes International) while, cathode and desalination compartments were separated by a cation exchange membrane (CMI7000, Membranes International). In order to keep membranes hydrated and expanded, pre-conditioning of each membrane was performed by immersing them in 85.6 mM NaCl solution at 40 °C for a day and washed with distilled water before to use. Carbon cloth was used as anode and cathode electrodes with an approximate surface area of 11.2 cm^2^. To remove the excess residues from carbon cloth, electrodes were washed with 1 N NaOH solution followed by 1 N HCl solution and finally washed and soaked in distilled water over night before their use as electrodes^56^. Both electrodes were inserted into the MDCs and were interconnected by a Titanium wire through a 1 KΩ external resistor. MDC configurations and connections were set following the procedures as reported in previous studies^12,17^.

NiA_mox_MDC, contained anammox bacteria as biocatalysts in the cathode compartment and air was supplied at 10 ml/min to achieve partial nitritation process (Fig. 7a). 70 mg/L of ammonium nitrogen and anammox media with trace elemental solutions I and II as described above were used as cathode solution in this MDC. 10 g/L of NaCl solution was added in all MDC desalination compartments. CMDC was operated as air cathode MDC, without anammox bacteria in cathode chamber (Fig. 7b). The CMDC cathode was supplied with an aeration rate of 10 ml/min with anammox media and trace elemental solutions I and II in it. AnA_mox_MDC contained anammox bacteria as biocatalysts in the cathode compartment and was operated under near anaerobic conditions to perform direct anammox process in the cathode compartment (Fig. 7c). 70 mg/L of ammonium, 70 mg/L of nitrite with anammox media and trace elemental solutions I and II were used as cathode solution in this MDC. All three MDCs were operated in fed-batch mode at a room temperature of ~22 °C. A total of eight cycles (n = 8) were run to study each process and make a comparison. COD, TDS, NH_4_^+^-N, NO_2_^−^-N, NO_3_^−^-N, pH, and, EC of samples were analyzed at the start and the end of each batch cycle. Fresh solutions of wastewater, anammox media and saline water were used in each fed-batch cycle.

### Analytical methods

COD concentrations were measured using the procedure in Standard Method for Examination of Water and Wastewater^57^. Total dissolved solids (TDS) concentrations and electrical conductivity of the solution were measured using a conductivity meter (Extech EC400). Colorimetric methods (Hach methods 8039 & 8114) were used to analyze the concentration of ammonium nitrogen (NH_4_^+^-N), nitrite nitrogen (NO_2_^−^-N) and nitrate nitrogen (NO_3_^−^-N).

### Calculations

Fluke (287 true RMS) multimeter was connected in each MDC to record the voltage drop (V) continuously across a 1-kΩ load resistor (R) at a 15-minute interval. Ohm’s law as in Eq. 1 was used to calculate the current across the 1 kΩ load resistor.

1 $${\rm{V}}={\rm{I}}\times {\rm{R}}$$

Power density (W/m^3^) was calculated using Eq. 2.

2 $${{\rm{P}}}_{{\rm{An}}}={\rm{R}}\times {{\rm{I}}}^{2}/{{\rm{V}}}_{{\rm{an}}}$$

where I is the current flowing through the resistor in ampere and V_an_ is the volume of the anode chamber in cubic meter.

COD removal efficiencies were calculated using Eq. 3

3 $$ \% \,{\rm{Removal}}\,{\rm{efficiency}}\,(\text{COD})=\frac{{{\rm{COD}}}_{{\rm{i}}}-{{\rm{COD}}}_{{\rm{f}}}}{{{\rm{COD}}}_{{\rm{i}}}}\times 100 \% $$

where COD_i_ and COD_f_ are the initial and final COD (mg/L) concentrations of the anode solution, respectively.

The calculation of coulombic efficiency (CE) was accompanied using Eq. 4

4 $${\rm{CE}}=({\rm{M}}{\int }_{0}^{{{\rm{t}}}_{{\rm{b}}}}{\rm{I}}\,{\rm{dt}})/({{\rm{FbV}}}_{{\rm{an}}}{\rm{\Delta }}\text{COD})$$

here, Faraday’s constant (96,485 C/mol) is denoted by F, anode liquid volume is denoted by V_an_, the number of moles of electrons generated per mole of substrate is denoted by b (b = 4), ΔCOD (g/L) is the concentration change in COD over the batch cycle time t_b_ and molecular weight of oxygen is denoted by M (M = 32). The desalination ratio (η) of the saline water was based on the change in the conductivity of saline water (the concentration of NaCl in this study showed a linear fit with its conductivity) over a batch cycle and was calculated using Eq. 5

5 $${\rm{\eta }}=\frac{{{\rm{\sigma }}}_{{\rm{i}}}-{{\rm{\sigma }}}_{{\rm{f}}}}{{{\rm{\sigma }}}_{{\rm{i}}}}\times 100 \% $$

where σ_i_ and σ_f_ are the initial and final conductivities of the saline water in the desalination compartment over a fed-batch cycle respectively.

The charge harvested (Q) was calculated by integrating current (I) over the desalination cycle (${\rm{Q}}={\int }_{0}^{{{\rm{t}}}_{{\rm{b}}}}{\rm{I}}\,{\rm{dt}}$) and the theoretical charge (Q_th_) was calculated as Q_th_ = 0.171 mol/L x η x F x V where, 0.171 mol/L is equivalent to initial NaCl concentration (10 g/L) in the desalination compartment, and V is the volume of the desalination compartment. The charge transfer efficiency of each MDC was calculated as the ratio of Q_th_/Q. This calculation was based on the assumption that one mole of electrons is required to remove one mole of NaCl. Energy produced by MDCs was calculated by using Eq. 6.

6 $${\rm{Energy}}\,{\rm{produced}}\,{\rm{by}}\,{\rm{MDC}}={\int }_{0}^{{{\rm{t}}}_{{\rm{b}}}}{\rm{IVdt}}$$

Energy consumption due to aeration was estimated based on an aeration efficiency of 1.2 kg O_2_/kWh^58,59^.

### Electrochemical test

Cyclic voltammetry (CV) test was performed at the turn over condition (a condition at which a stable voltage generation is observed) of each MDC. The CV test was conducted to compare the redox activities and biofilm performances in AnA_mox_MDC, NiA_mox_MDC and CMDC. The test was conducted with a three-electrode (working, counter and reference electrode) cell system: for analysis of anodic biofilm, anode was used as working electrode, Ag/AgCl (RE 5B, Basi) as a reference electrode and cathode as counter electrode while, for the analysis of cathodic biofilm, cathode was used as working electrode and anode as counter electrode. Reference electrode was always placed close to the working electrode. For the anodic CV tests, scanned potential was varied between −0.6 V and +0.5 V at a scan rate of 1 mV/s by using Gamry 1010e potentiostat, while, for cathodic CV, scanned potential ranged from −0.5 V to +0.5 V^12^. Electrochemical Impedance Spectroscopy (EIS) was performed to investigate any differences in electrochemical performance and ohmic resistance of NiA_mox_MDC and AnA_mox_MDC at an open circuit voltage of each MDC at the end of the cycle. EIS measurements were conducted by using Gamry 1010e potentiostat with 10 mV amplitude ac signal at frequencies between 10^5^ and 0.01 Hz^60^.

### Statistical analysis

One-way analysis of variance test (ANOVA) was performed to obtain the statistical significance of differences using the statistical analysis system, SAS 9.4. A probability value, *P* < 0.01, was considered significant.

## Supplementary information

Accomplishing a N-E-W synergy
