# Supplementary material for: Accomplishing a N-E-W (nutrient-energy-water) synergy in a bioelectrochemical nitritation-anammox process
Source: Sci Rep. 2019 Jun 24;9:9201. doi: 10.1038/s41598-019-45620-2 (PMC6591234; doi:10.1038/s41598-019-45620-2)
Supplement: Supplementary file 1 — Accomplishing a N-E-W synergy [file 41598_2019_45620_MOESM1_ESM.docx]

**Supplementary Information**

**Accomplishing a N-E-W (nutrient-energy-water) synergy in a bioelectrochemical nitritation-anammox process**

Umesh Ghimire, Veera Gnaneswar Gude*

Department of Civil and Environmental Engineering

Mississippi State University, Mississippi State, MS 39762.

*Correspondence and requests for materials should be addressed to Gude, V.G. ([gude@cee.msstate.edu](mailto:gude@cee.msstate.edu))

No. of Pages – **9**

No. of Tables - **5**

No. of Figures - **3**

**Supplementary information:**

**Supplementary Table S1**. Maximum voltage and power density of each MDC at different batch cycles.

**Supplementary Table S2.** Ammonium removal at cathode chamber in each cycle of different MDCs.

**Supplementary Table S3**. Comparison of bioelectrochemical nitritation process with traditional anammox nitritation process.

**Supplementary Table S4**. Charge transfer efficiency of each MDC in different batch cycles.

**Supplementary Table S5**: Summary of energy production and consumption in different MDCS.

**Supplementary Figure S1:** Total nitrogen removal in eight batch cycles: (A) NiA_mox_MDC; (B) AnA_mox_MDC: AND (C) CMDC.

**Supplementary Figure S2**. Average charge transfer efficiency of different MDCs.

**Supplementary Figure S3**: Box plot for (A) distribution of COD, (B) distribution of CE, (C) distribution of TDS.

**Supplementary Table S1. Maximum voltage and power density of each MDC at different batch cycles**

**A. Air flow rate of 10 ml/min**

| MDCs | Maximum voltage (mV) | | | | | Maximum power density (mW/m^3^) | | | | |
| --- | --- | --- | --- | --- | --- | --- | --- | --- | --- | --- |
|  | Start up | 1^st^ cycle | 2^nd^ cycle | 3^rd^ cycle | 4^th^ cycle | Start up | 1^st^ cycle | 2^nd^ cycle | 3^rd^ cycle | 4^th^ cycle |
| CMDC | 40.8 | 188.7 | 206.3 | 238.9 | 240.3 | 27.7 | 593.4 | 709.3 | 951.2 | 962.5 |
| AnA_mox_MDC | 37.2 | 108.7 | 179 | 163 | 168.2 | 23 | 534 | 442.9 | 442.9 | 471.5 |
| NiA_mox_MDC | 26.5 | 160.32 | 212.1 | 236.3 | 255.2 | 11.70 | 749.77 | 930.6 | 930.6 | 1085.4 |

**B. Air flow rate of 3 ml/min**

| MDCs | Maximum voltage (mV) | | | | Maximum power density (mW/m^3^) | | | |
| --- | --- | --- | --- | --- | --- | --- | --- | --- |
|  | 5^th^ cycle | 6^th^ cycle | 7^th^ cycle | 8^th^ cycle | 5^th^ cycle | 6^th^ cycle | 7^th^ cycle | 8^th^ cycle |
| CMDC | 207.1 | 209.6 | 209.9 | 209.4 | 714.8 | 732.2 | 734.3 | 730.8 |
| AnA_mox_MDC | 179.9 | 168.9 | 179 | 183.2 | 539.4 | 475.4 | 534 | 558.1 |
| NiA_mox_MDC | 243.9 | 240.7 | 238.9 | 242.7 | 991.4 | 965.6 | 951.2 | 981.7 |

**Supplementary Table S2. Ammonium removal at cathode chamber in each cycle of different MDCs**

**A. Air flow rate of 10 ml/min**

| MDCs | Ammonium (mg/L) | | | | | | | | | | | |
| --- | --- | --- | --- | --- | --- | --- | --- | --- | --- | --- | --- | --- |
|  | 1^st^ cycle | | | 2^nd^ cycle | | | 3^rd^ cycle | | | 4^th^ cycle | | |
|  | Initial | Final | Removal (%) | Initial | Final | Removal (%) | Initial | Final | Removal (%) | Initial | Final | Removal (%) |
| CMDC | 72 | 18 | 75 | 70 | 17.5 | 75 | 72 | 16.3 | 77.3 | 68 | 18 | 73.5 |
| AnA_mox_MDC | 71 | 16.5 | 76.7 | 70 | 14.3 | 79.5 | 71 | 13.0 | 81.6 | 70 | 10.8 | 85.6 |
| NiA_mox_MDC | 72 | 1.2 | 98.3 | 71 | 1.5 | 97.8 | 68 | 10.3 | 84.8 | 72 | 15.36 | 78.6 |

**B. Air flow rate of 3 ml/min**

| MDCs | Ammonium (mg/L) | | | | | | | | | | | |
| --- | --- | --- | --- | --- | --- | --- | --- | --- | --- | --- | --- | --- |
|  | 5^th^ cycle | | | 6^th^ cycle | | | 7^th^ cycle | | | 8^th^ cycle | | |
|  | Initial | Final | Removal (%) | Initial | Final | Removal (%) | Initial | Final | Removal (%) | Initial | Final | Removal (%) |
| CMDC | 70 | 14.2 | 79.8 | 70 | 14.2 | 79.7 | 70 | 14.0 | 80.0 | 68 | 13.0 | 80.8 |
| AnA_mox_MDC | 72 | 10 | 86.1 | 72 | 9.8 | 86.4 | 71 | 9.32 | 86.9 | 70 | 10.6 | 84.8 |
| NiA_mox_MDC | 69 | 1.3 | 98.3 | 71 | 1.5 | 97.8 | 69 | 10.3 | 84.8 | 72 | 15.36 | 78.6 |

**Supplementary Table S3. Comparison of bioelectrochemical nitritation process with traditional anammox nitritation process.**

| S.N | Process | Reactor volume  (L) | HRT  (hrs) | COD removal efficiency (%) | TN removal efficiency (%) | Energy consumption (kW-hr/m^3^) | Energy production (kW-hr/m^3^) | Reference |
| --- | --- | --- | --- | --- | --- | --- | --- | --- |
| 1. | Bioelectrochemical nitration anammox process | 0.06 | 72 | 72.1 | 70.0 | 0.0221 | 0.049 | This study |
| 2 | Nitritation anammox MBR | 4 | 12 | 96 | 81.0 | 0.09 | none | ^1^ |
| 3 | Nitritation/anammox process | NR | 2 | 48 | 15 | 0.021 | none- | ^2^ |

MBR = membrane bio reactor NR = not reported

**Supplementary Table S4. Charge transfer efficiency of each MDC in different batch cycles**

**A. Air flow rate of 10 ml/min**

| MDCs | Charge transfer efficiency (%) |  |  |  |  |
| --- | --- | --- | --- | --- | --- |
|  | Start up | 1^st^ cycle | 2^nd^ cycle | 3^rd^ cycle | 4^th^ cycle |
| CMDC | 210.2 | 427.3 | 418.7 | 454.9 | 359.9 |
| AnA_mox_MDC | 185.2 | 468.1 | 511.1 | 504.5 | 392 |
| NiA_mox_MDC | 212.3 | 573.3 | 591.2 | 557.2 | 430.4 |

**B. Air flow rate of 3 ml/min**

| MDCs | Charge transfer efficiency (%) | | | |
| --- | --- | --- | --- | --- |
|  | 5^th^ cycle | 6^th^ cycle | 7^th^ cycle | 8^th^ cycle |
| CMDC | 359.9 | 676.4 | 780.7 | 754 |
| AnA_mox_MDC | 490 | 854.4 | 571 | 609.7 |
| NiA_mox_MDC | 431.4 | 553.1 | 668.9 | 901.8 |

**Supplementary Table S5. Summary of energy production and consumption in different MDCs**

1. **Air flow rate of 10 ml/min**

| MDCs | Start up | | | 1^st^ cycle | | | 2^nd^ cycle | | | 3^rd^ cycle | | | 4^th^ cycle | | |
| --- | --- | --- | --- | --- | --- | --- | --- | --- | --- | --- | --- | --- | --- | --- | --- |
|  | Energy produced (kW-hr/m^3^) | Energy consumed by air pump (kW-hr/m^3^) | Net energy^a^ | Energy produced (kW-hr/m^3^) | Energy consumed by air pump (kW-hr/m^3^) | Net energy | Energy produced (kW-hr/m^3^) | Energy consumed by air pump (kW-hr/m^3^) | Net energy | Energy produced (kW-hr/m^3^) | Energy consumed by air pump (kW-hr/m^3^) | Net energy | Energy produced (kW-hr/m^3^) | Energy consumed by air pump (kW-hr/m^3^) | Net energy |
| CMDC | 0.000412 | 0.027 | -0.026 | 0.036 | 0.021 | +0.015 | 0.033 | 0.015 | +0.018 | 0.043 | 0.021 | +0.022 | 0.0481 | 0.027 | +0.0211 |
| AnA_mox_MDC | 0.000480 | 0 | +0.000480 | 0.01 | 0 | +0.01 | 0.0213 | 0 | +0.0213 | 0.0301 | 0 | +0.0301 | 0.0213 | 0 | +0.0213 |
| NiA_mox_MDC | 0.0000879 | 0.021 | -0.026 | 0.0221 | 0.021 | 0 | 0.040 | 0.015 | +0.025 | 0.056 | 0.021 | +0.035 | 0.0579 | 0.027 | +0.0309 |

**B. Air flow rate of 3 ml/min**

| MDCs | 5^th^ cycle | | | 6^th^ cycle | | | 7^th^ cycle | | | 8^th^ cycle | | |
| --- | --- | --- | --- | --- | --- | --- | --- | --- | --- | --- | --- | --- |
|  | Energy produced (kW-hr/m^3^) | Energy consumed by air pump (kW-hr/m^3^) | Net energy | Energy produced (kW-hr/m^3^) | Energy consumed by air pump (kW-hr/m^3^) | Net energy | Energy produced (kW-hr/m^3^) | Energy consumed by air pump (kW-hr/m^3^) | Net energy | Energy produced (kW-hr/m^3^) | Energy consumed by air pump (kW-hr/m^3^) | Net energy |
| CMDC | 0.027 | 0.004 | +0.023 | 0.024 | 0.015 | +0.018 | 0.043 | 0.021 | +0.022 | 0.0481 | 0.027 | +0.0211 |
| AnA_mox_MDC | 0.05 | 0 | +0.05 | 0.035 | 0 | +0.0213 | 0.0301 | 0 | +0.0301 | 0.0213 | 0 | +0.0213 |
| NiA_mox_MDC | 0.05 | 0.004 | 0.046 | 0.035 | 0.015 | +0.025 | 0.056 | 0.021 | +0.035 | 0.0579 | 0.027 | +0.0309 |

^a^Negative (-) indicates energy-negative MDC and positive (+) indicates energy-positive MDC


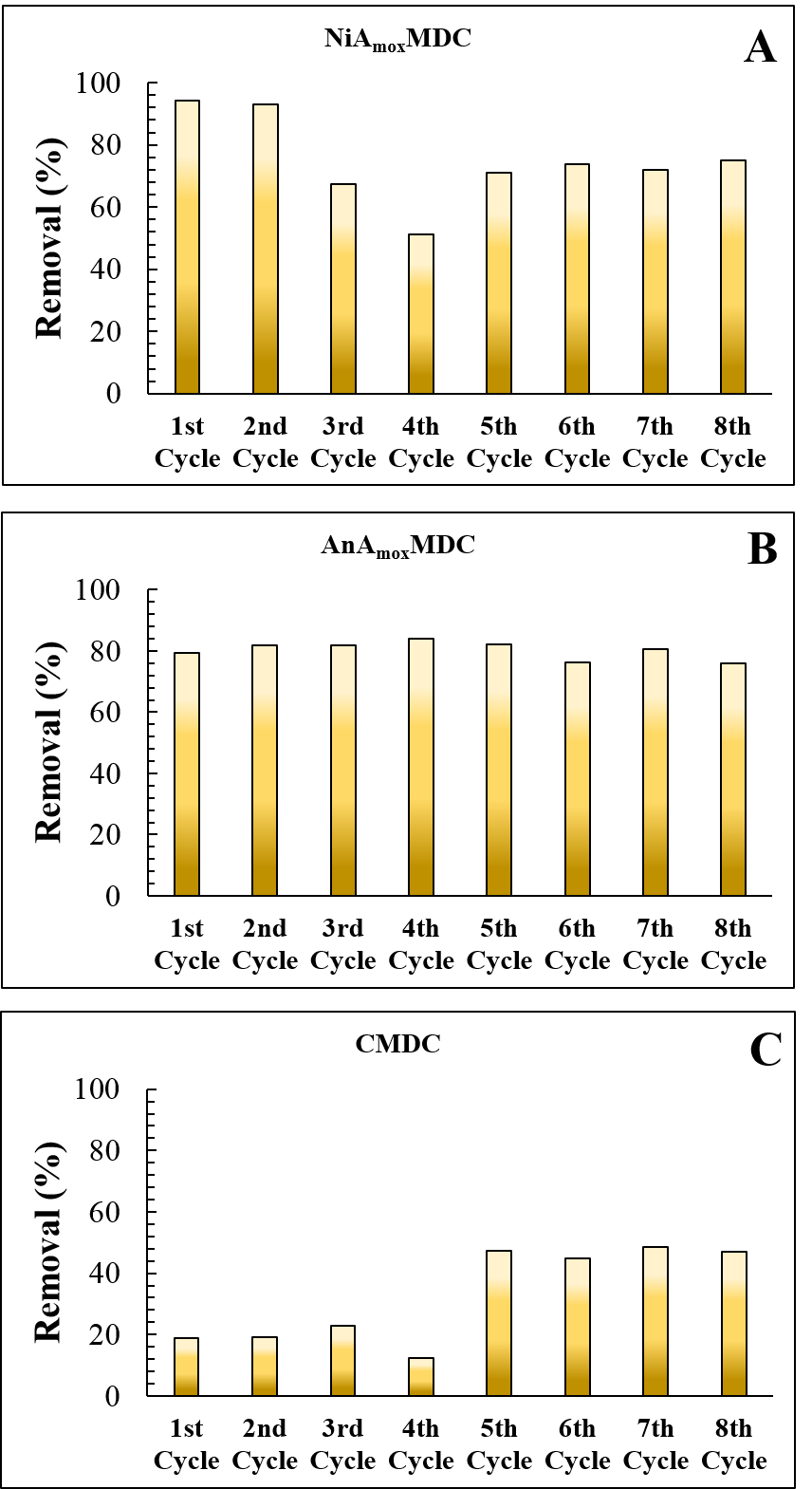


Supplementary Figure S1: Total nitrogen removal in eight batch cycles: (A) NiA_mox_MDC; (B) AnA_mox_MDC: and (C) CMDC.


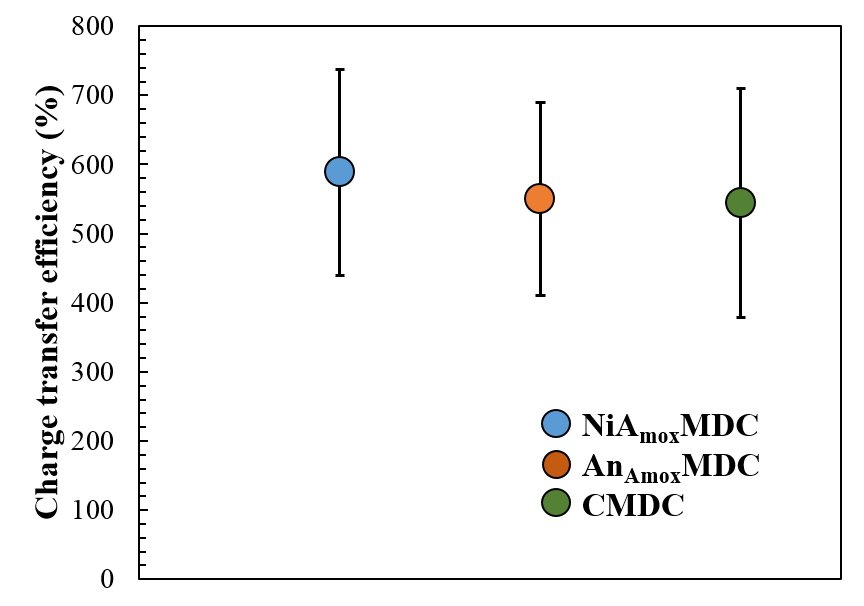


Supplementary Figure S2: Average charge transfer efficiency of different MDCs


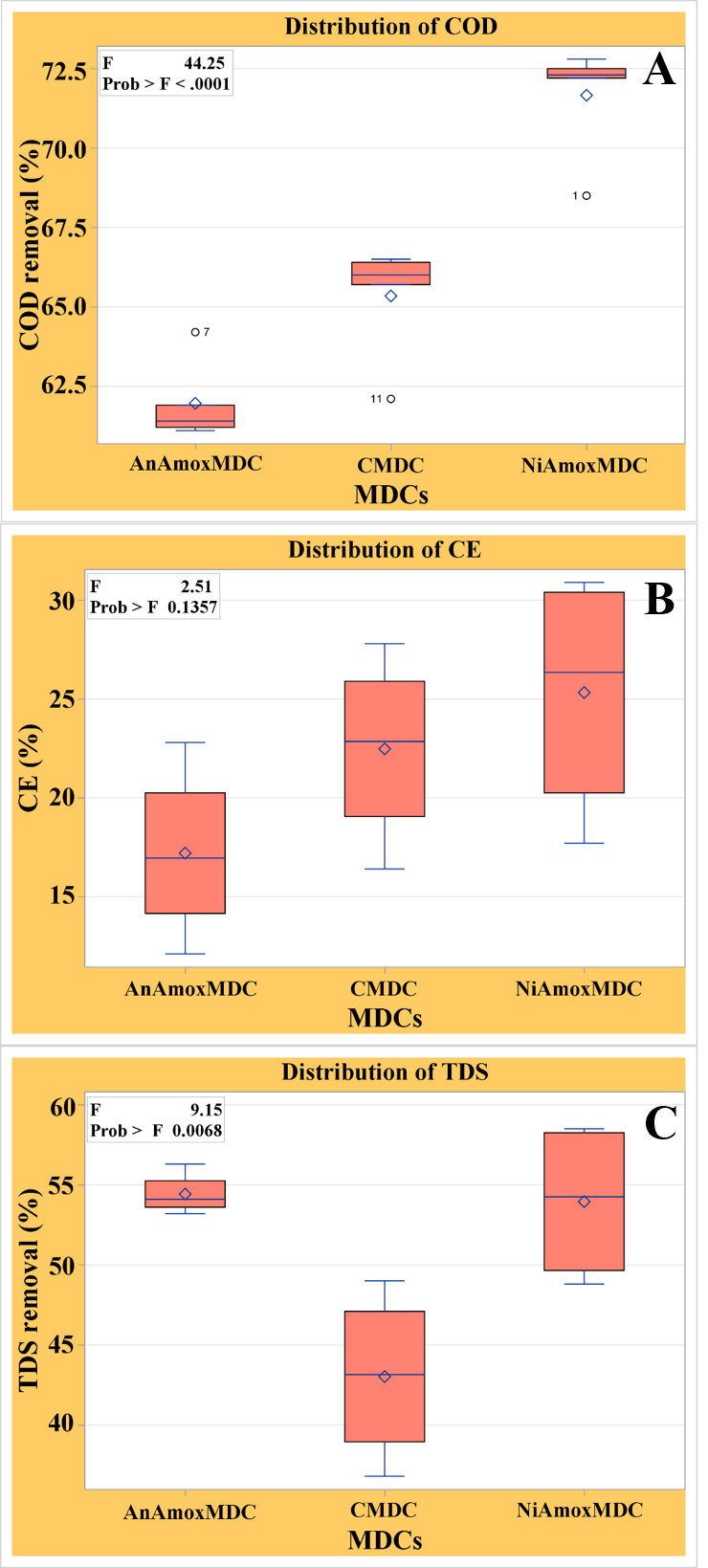


Supplementary Figure S3: Box plot for (A) distribution of COD, (B) distribution of CE, (C) distribution of TDS.

References

1. Dai, W., Xu, X., Liu, B. & Yang, F. Toward energy-neutral wastewater treatment: A membrane combined process of anaerobic digestion and nitritation–anammox for biogas recovery and nitrogen removal. *Chem. Eng. J.* **279,** 725–734 (2015).

2. Siegrist, H., Salzgeber, D., Eugster, J. & Joss, A. Anammox brings WWTP closer to energy autarky due to increased biogas production and reduced aeration energy for N-removal. *Water Sci. Technol.* **57,** 383–388 (2008).
